# Variability in sperm form and function in the context of sperm competition risk in two *Tupinambis* lizards

**DOI:** 10.1002/ece3.1262

**Published:** 2014-10-07

**Authors:** Cecilia S Blengini, Naretto Sergio, Cardozo Gabriela, Laura C Giojalas, Chiaraviglio Margarita

**Affiliations:** 1 Laboratorio de Biología del Comportamiento, Facultad de Ciencias Exactas, Físicas y Naturales, Instituto de Diversidad y Ecología Animal (IDEA) CONICET, Universidad Nacional de CórdobaAv. Vélez Sársfield 299, X5000JJC, Córdoba, Argentina; 2 Centro de Biología Celular y Molecular, Facultad de Ciencias Exactas, Físicas y Naturales, Instituto de Investigaciones Biológicas y Tecnológicas (IIByT) CONICET, Universidad Nacional de CórdobaAv.Velez Sarsfield 1611, X5016GCA, Córdoba, Argentina

**Keywords:** Postcopulatory sexual selection, sperm evolution, sperm morphometry, sperm velocity, Squamata

## Abstract

In polyandrous species, sperm morphometry and sperm velocity are under strong sexual selection. Although several hypotheses have been proposed to explain the role of sperm competition in sperm trait variation, this aspect is still poorly understood. It has been suggested that an increase in sperm competition pressure could reduce sperm size variation or produce a diversity of sperm to maximize male fertilization success. We aim at elucidating the variability of sperm morphometric traits and velocity in two *Tupinambis* lizards in the context of sperm competition risk. Sperm traits showed substantial variation at all levels examined: between species, among males within species, and within the ejaculate of individual males. Sperm velocity was found to be positively correlated with flagellum: midpiece ratio, with relatively longer flagella associated with faster sperm. Our results document high variability in sperm form and function in lizards.

## Introduction

Polyandry can promote the spatial and temporal overlap of ejaculates from multiple males within the female reproductive tract, promoting postcopulatory sexual selection and male adaptations that ensure success during sperm competition (Parker 1970; Snook 2005; Pizzari and Parker 2009). In taxa with high risk of sperm competition, females are more likely to mate with multiple males within a single reproductive cycle (Snook and Pizzari 2012), and males need to invest more energy in ejaculate traits (Parker 1998). Because sperm competition often favors the evolution of larger testes, relative testis mass is considered a reliable index of sperm competition risk (Tourmente et al. 2009, 2013; Snook and Pizzari 2012).

Sperm morphometry is under strong sexual selection and has been shown to evolve rapidly, with sperm having a variety of sizes and shapes both between and within species (Pitnick et al. 2009a). Sperm velocity is an important determinant of male fertility in noncompetitive (Malo et al. 2005; Gomendio et al. 2007; Gomendio and Roldan 2008) and competitive contexts (Birkhead et al. 1999; Gage et al. 2004; Gomendio and Roldan 2008; Pizzari and Parker 2009). Selection is expected to act on sperm form and function, but the evidence supporting this assumption remains controversial (Simmons and Fitzpatrick 2012; Gillies et al. 2013; Simpson et al. 2013).

Interspecific studies have found that sperm competition exerts directional selection for an increase in sperm size (Gomendio et al. 2007; Fitzpatrick et al. 2009; Lüpold et al. 2009a; Tourmente et al. 2011a) and sperm velocity (Lüpold et al. 2009a; Tourmente et al. 2011a). Because the structure and function of sperm components vary among taxa, sperm competition might have different effects on various components of sperm among taxa (Johnson and Briskie 1999; Gomendio and Roldan 2008; Tourmente et al. 2009, 2011a). Moreover, a positive relationship between total sperm size and swimming velocity was found in different taxa (Gomendio and Roldan 2008; Fitzpatrick et al. 2009; Tourmente et al. 2011a). This relationship is not clear at the intraspecific level, suggesting that the selective force may operate differently at the macro- and micro-evolutionary levels (Gomendio and Roldan 2008).

At the intraspecific level, postcopulatory sexual selection may play an important role in regulating among- and within-male sperm size variation (Calhim et al. 2007; Kleven et al. 2007; Immler et al. 2008; Helfenstein et al. 2010). On the one hand, an increase in sperm competition pressure might reduce among- and within-male variation in sperm size (Birkhead et al. 2005; Calhim et al. 2007; Kleven et al. 2007; Immler et al. 2008; Lifjeld et al. 2013; Van der Horst and Maree 2014) toward an optimal sperm design (Calhim et al. 2007). On the other hand, recent studies suggest that variation in sperm morphometry persists and may be an important determinant of relative reproductive fitness (Crean and Marshall 2008; Morrow et al. 2008; Immler et al. 2010; Bakker et al. 2014). Calhim et al. (2011) showed that variation can be maintained despite extreme promiscuity. Moreover, Helfenstein et al. (2010) proposed that an individual male may produce a diversity of sperm to maximize fertilization success in the context of sperm competition.

Recent studies focusing on within-male variation between ejaculates suggest that sperm morphometry (Immler et al. 2010; Calhim et al. 2011) and velocity (Lüpold et al. 2012) can be phenotypically plastic traits that can be adjusted to social environments. Moreover, there are evidences of within-ejaculate sperm size variation (Malo et al. 2006; Schulte-Hostedde and Montgomerie 2006; Helfenstein et al. 2010; Immler et al. 2010; Calhim et al. 2011; Lüpold et al. 2012; Lifjeld et al. 2013; Bakker et al. 2014; Van der Horst and Maree 2014). It has been argued that within-male variation in sperm size may represent developmental noise (Parker and Begon 1993; Hellriegel and Blanckenhorn 2002) or be influenced by male condition (Schulte-Hostedde and Montgomerie 2006). However, within-ejaculate sperm variation could be important to understand sperm diversification, which may be associated with variation in sperm function (Immler et al. 2010; Simpson et al. 2013). Phenotypic variation within individual ejaculates may be the result of sexual selection pressures; males may produce different specialized sperm, each one aiming at a different optimum (Pizzari and Parker 2009).

Because swimming speed is the result of the combination of different sperm components, ratios between the dimensions of different components seem better at explaining sperm swimming velocity than a single component (Gomendio and Roldan 2008; Humphries et al. 2008; Lüpold et al. 2009a; Fitzpatrick et al. 2010). Although several intraspecific studies have found no significant association between sperm size and velocity (Fitzpatrick et al. 2009; Lüpold et al. 2009b), other works have provided contrasting evidences (see Malo et al. 2006; Firman and Simmons 2010; Helfenstein et al. 2010). Recent studies showed the importance of taking into account the within-male variation in sperm traits to find the relationship between sperm morphometry and velocity (Fitzpatrick et al. 2010; Simpson et al. 2013).

Although a few studies have addressed sperm competition in Squamata (lizards and snakes) (Birkhead and Møller 1993; Olsson and Madsen 1998; Schulte-Hostedde and Montgomerie 2006; Tourmente et al. 2009), the mechanisms underlying success in sperm competition are not well understood. Most species of Squamata are polygynandrous (Duvall et al. 1992; Vitt and Caldwell 2009), that is, they exhibit a multi-male, multi-female polygamous mating system. Thus, there is ample opportunity for sperm competition arising from female matings with multiple partners within each ovarian cycle (Olsson and Madsen 1998; Zamudio and Sinervo 2000; Laloi et al. 2004). In some species, there is evidence of multiple paternity (Olsson and Madsen 1998; Calsbeek et al. 2007; Keogh et al. 2013), and females possess sperm storage structures (Sever and Hamlett 2002). However, the variability of sperm traits in relation to sperm competition risk has not been addressed in lizards.

**Figure 1:**
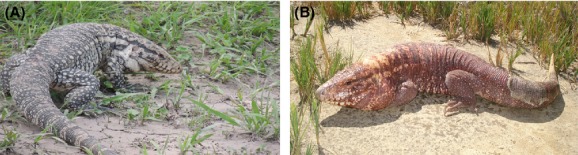
Males of *Tupinambis* lizards. (A) *Tupinambis merianae*; (B) *Tupinambis rufescens*.

Here we focused on two sister species, *Tupinambis merianae* and *T. rufescens* (Cabaña et al. 2014) (Fig. 1), which are phenotypically similar, share ecological similarities (Cardozo et al. 2012), and exhibit sexual size dimorphism, with males larger than females. Moreover, males of these two species present sexual dimorphism in jaw muscle, a secondary sexual character that could be influenced by inter and intrasexual selection (Naretto et al. 2014). Furthermore, in both species, sex ratio is biased to males, especially in *T. rufescens,* suggesting that these species are exposed to different contexts of competition (Naretto et al. 2014). Hence, interpreting the variation in sperm traits in relation to sperm competition risk in two sister species may contribute to our understanding of selective pressures acting on sperm evolution.

We aim at elucidating the variability of sperm morphometric and dynamic traits in the context of sperm competition risk in *Tupinambis* lizards. We quantified the variation in sperm traits between species, and among and within males in each species. Furthermore, we show the relationship between sperm morphometry and velocity in lizards.

## Methods

### Study species

*Tupinambis merianae* and *T. rufescens* breed seasonally in spring (Fitzgerald et al. 1993). These lizard species are included in the Appendix II of the Convention on International Trade of Endangered Species of Wild Fauna and Flora (CITES 2008); in Argentina, these lizards are under legal commercial harvesting (Porini 2006).

### Data collection

*Tupinambis* individuals were caught by local authorized hunters from wild populations in central Argentina (*T. rufescens*: 29°35′W, 64°10′S to 31°10′W, 63°15′S and *T. merianae*: 30°55′W, 63°40′S to 31°45′W, 62°15′S) throughout reproductive season, October to December (Naretto et al. 2014). Then, even individuals with low probability of capture are more likely to be trapped eventually (Biro 2013). We are authorized by the government environmental agencies for scientific capture, and we selected and accompanied local hunters to standardize the sampling protocol with the aim of avoiding size bias in capture rates. Specimens were killed for the legal skin trade, in accordance with AVMA Guidelines on Euthanasia (AVMA 2007). We weighed body mass and the mass of both testes of all individuals.

### Sperm sampling procedure

Specimens were dissected and spermatozoa were obtained from the terminal portion of the epididymis (Depeiges and Dacheux 1985). Time elapsed between animal death and sperm analyses ranged from 2 to 3 h. During this period, sperm sample was not affected because the average percentage of progressive sperm was 93.30 ± 4.81% in *T. merianae* and 96.03 ± 3.72% in *T. rufescens*. Moreover, the viability of sperm sample was higher than 80% for both species.

All the samples obtained were collected in a 1.5 mL plastic tube containing approximately 90 *μ*L of phosphate buffered saline (PBS). Sperm concentration was estimated using a Neubauer chamber, and the samples were diluted to a concentration of 1.10^6^ cells/mL in Biggers, Whitten, and Wittingham culture medium (Biggers et al. 1971) supplemented with 4% bovine serum albumin, prior to observation (Tourmente et al. 2011b).

### Sperm morphometry

We obtained sperm morphometric data from 74 males of *Tupinambis merianae* and 43 individuals of *T. rufescens* (Table 1). Aliquots of sperm samples were fixed for photography in 2% formaldehyde (Tourmente et al. 2009) and stained with Blue Brilliant Coomassie (Firman and Simmons 2010). The samples were examined at 400× magnification under a phase contrast Nikon eclipse Ti microscope (Nikon Instruments Inc, Tokyo, Japan). Microphotographs of the samples were taken using Nikon DS-Qi1Mc digital camera with a controller DS-U2 (Nikon Instruments Inc). Absolute length (*μ*m) of head, midpiece and flagellum, and total sperm length of 50 spermatozoa per individual was measured using software Image J version 1.43u (NIH, Bethesda, MD).Then, the ratios of flagellum: head length, flagellum: midpiece length, and head: midpiece were estimated. All measurements were made by the same person to reduce potential interobserver variability. Mean trait values for each species were calculated from the means from each individual of that species.

**Table 1:** Comparison of sperm traits between *Tupinambis merianae* and *T. rufescens*

Sperm traits	*T. merianae*	*T. rufescens*	Statistics	*P*-value
Head length (*μ*m)	13.64 ± 0.75^a^	13.28 ± 1.04	*F* = 4.20	0.0428
	5.5^b^	7.83		
Midpiece length (*μ*m)	5.19 ± 0.29	4.94 ± 0.38	*F* = 17.24	<0.0001
	5.6	7.66		
Flagellum length (*μ*m)	59.97 ± 1.23	57.69 ± 1.19	*F* = 94.11	<0.0001
	2.05	2.05		
Total sperm length (*μ*m)	79.19 ± 1.22	76.32 ± 1.69	*F* = 109.70	<0.0001
	1.55	2.21		
Curvilinear velocity (*μ*m/sec)	31.52 ± 6.61	34.99 ± 7.5	*F* = 5.83	0.0172
	20.97	22.93		
Straight velocity (*μ*m/sec)	24.79 ± 5.63	27.79 ± 6.65	*F* = 5.03	0.0267
	22.71	24.03		
Linearity	0.78 ± 0.05	0.78 ± 0.04	*F* = 0.00061	0.9804
	5.89	5.18		
CV_wm_ head length	7.24 ± 2.24	8.01 ± 2.62	*H* = 3.40	0.0651
CV_wm_ midpiece length	9.71 ± 2.01	11.57 ± 2.35	*H* = 15.93	<0.0001
CV_wm_ flagellum length	5.34 ± 1.91	5.76 ± 2.54	*H* = 0.42	0.5160
CV_wm_ total sperm length	4.23 ± 1.44	4.7 ± 2.34	*H* = 0.55	0.4574
CV_wm_ curvilinear velocity	20.87 ± 3.98	20.90 ± 4.79	*H* = 0.04	0.8320
CV_wm_ straight velocity	31.46 ± 6.60	30.81 ± 6.40	*H* = 0.07	0.7872
CV_wm_ linearity	18.90 ± 5.22	16.83 ± 3.84	*H* = 3.92	0.0476

a Mean ± SD;

b CV among males

### Sperm dynamic traits

We obtained sperm dynamics data from 82 males of *Tupinambis merianae* and 39 individuals of *T. rufescens* (Table 1). Aliquots (500 *μ*L) of sperm sample were incubated at 25°C in thermally stable water baths for 30 min (Tourmente et al. 2011b). The sperm suspension (20 *μ*L) was placed in a plastic observation chamber and covered with a coverslip. Dynamic parameters were measured at room temperature (25°C) using a video microscopy system composed of a phase contrast microscope (CX41; Olympus, Tokyo, Japan) equipped with a video camera (ICAM 1500; Labomed, Fremont, CA). The software used to capture the digital videos was Virtualdub v.1.6.16. The samples were recorded at 100× magnification for 4 min with a random change of the microscope field every 5 sec. Subsequently, individual sperm tracks were followed for 3 sec in 45 cells/sample and transformed to a matrix of Cartesian coordinates using ImageJ version 1.43u (NIH) and its plug-in MtrackJ v. 1.1.0 (Eric Meijering). The following sperm dynamic parameters were calculated from this matrix using Spermtrack v. 4.2 (Universidad Nacional de Cordoba, Argentina): straight line velocity (VSL; *μ*m/sec), curvilinear velocity (VCL; *μ*m/sec), and linearity (LIN; LIN = VSL/VCL) (Blengini et al. 2011). Mean trait values for each species were calculated from the means of each individual of that species.

### Statistical analyses

The difference between species in relative testis mass (testis mass relative to body mass) was determined by an analysis of covariance (ANCOVA), using body mass as a covariable. As we observed that relative testis mass varied over the breeding season (*T. merianae n* = 92; *F* = 10.85, *P* < 0.0001*; T. rufescens n* = 45 *F* = 2.90, *P* = 0.0260), then, we considered testis data only from lizards collected during the peak of the breeding season for each species. Testis mass and body mass were log10 transformed.

To quantify among- and within-male variation in sperm traits, we calculated the coefficient of variation (CV) of sperm morphometric and dynamic traits for each male and calculated the mean for each trait from all males per species (Calhim et al. 2007). Statistical differences in the mean of sperm traits and within-male variation between species were determined by one-way nested ANOVA and Kruskal–Wallis, respectively. Moreover, in each species, differences in sperm traits among males were also determined by the nonparametric Kruskal–Wallis test. To test whether within-male variation of sperm traits was associated with testis development, we compared within-male variation of sperm traits among months during the breeding season (October–December) based on changes in testis mass over this period, using the nonparametric Kruskal–Wallis test. These statistical tests were conducted using InfoStat software (version 2012; Universidad de Cordoba, Argentina).

We used random models with restricted maximum likelihood parameter estimation function (REML) to partition total variance into variance between species and among and within males of a single species, and to estimate within-male and within-sperm (measurement) repeatability. To determine differences in within- male variation in sperm traits among males of each species, we ran two different models, one which initially assumed a common variance for individuals within each species, and the other in which variance was allowed to differ among individuals. The two models were compared using a likelihood ratio test to determine whether the intra-individual variance was significantly different for individuals of each species, following AIC criteria. These statistical tests were conducted using the software R (version 2.13.0; The R Foundation for Statistical Computing 2011).

To test whether sperm length was associated with sperm velocity, we performed multiple regression analysis using head length, midpiece length, and flagellum length for each species as predictors. The colinearity of sperm morphometric traits was discarded previously. Furthermore, to test whether within-male variation of sperm morphometry was associated with sperm velocity, we also performed multiple regression analysis using CV in head length, CV in midpiece length and CV in flagellum length for each species as predictors. We also performed a single regression analysis with VCL, VSL as dependent variables, and flagellum: head length ratio, flagellum: midpiece length ratio and head: midpiece length ratio as predictors. These statistical tests were performed using InfoStat software (version 2012; Universidad de Cordoba, Argentina).

## Results

We found that *Tupinambis rufescens* had greater relative testis mass (ANCOVA *F*_1,117_ = 5.17; *P* = 0.0248) than *T. merianae*. Then, we explored interspecific sperm variation in the two species. *T. merianae*, had all sperm components longer than *T. rufescens*. However, *T. rufescens* presented higher sperm velocity than *T. merianae* (Table 1). Furthermore, no differences in the pattern of movement were observed between species (Table 1).

**Figure 2:**
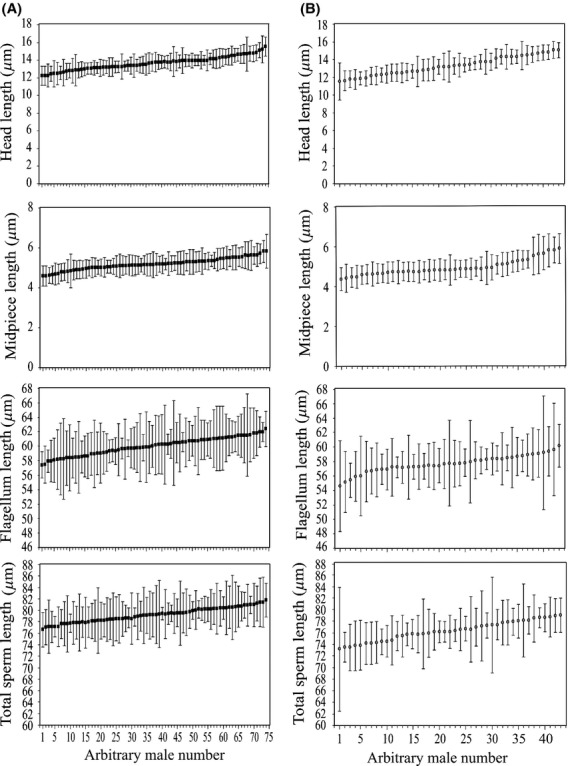
Within- and among-male variation in sperm morphometric traits in *Tupinambis* lizards. (A) *Tupinambis merianae* (black squares); (B) *Tupinambis rufescens* (gray dots). Squares and dots represent individual mean lengths (±standard deviation) ranked in order of magnitude.

**Figure 3:**
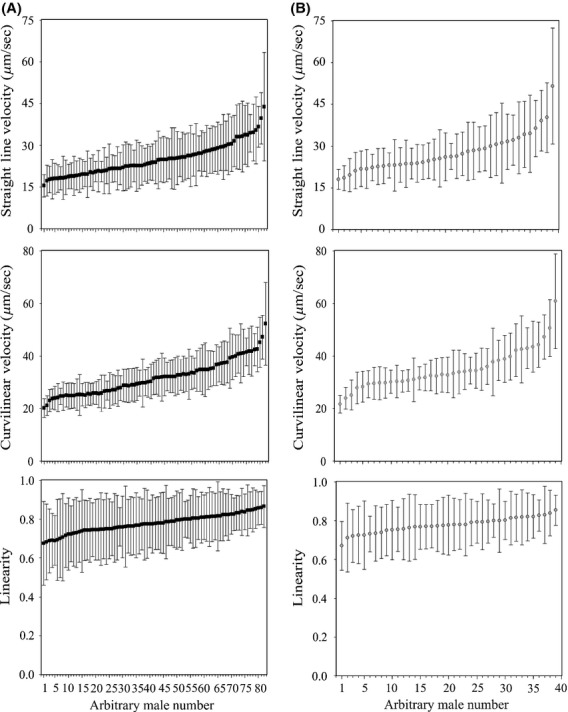
Within- and among-male variation in sperm dynamic traits in *Tupinambis* lizards. (A) *Tupinambis merianae* (black squares); (B) *Tupinambis rufescens* (gray dots). Squares and dots represent individual mean lengths (±standard deviation) ranked in order of magnitude.

We also studied the variation in sperm traits among and within males of each species. Significant differences among males were found in all sperm morphometric and dynamic traits in both species (*T. merianae*, head length: *H* = 1544.95, *P* < 0.0001; midpiece length: *H* = 969.65, *P* < 0.0001; flagellum length: *H* = 1068.37, *P* < 0.0001; total sperm length: *H* = 985.74, *P* < 0.0001; VCL: *H* = 1749.09, *P* < 0.0001; VSL: *H* = 1128.71, *P* < 0.0001; and LIN: *H* = 363.69, *P* < 0.0001. *T. rufescens*, head: *H* = 1161.6, *P* < 0.0001; midpiece: *H* = 622.68, *P* < 0.0001; flagellum: *H* = 465.4, *P* < 0.0001; total sperm length: *H* = 687.81, *P* < 0.0001; VCL: *H* = 787.23, *P* < 0.0001; VSL: *H* = 508.07, *P* < 0.0001; and LIN: *H* = 167.01, *P* < 0.0001*,* Figs. 2, 3). However, within-male variation was always the highest source of variation of sperm traits in both species, ranging from 58% (head length) to 76% (flagellum length) of the total of the variance in sperm morphometric traits (Table 2, Fig. 2) and from 51% (VCL) to 94% (LIN) of the total of the variance in sperm dynamic traits (Table 2, Fig. 3). Furthermore, we found high repeatability of the measurements in all sperm traits measured, which ranged from 0.84 to 0.99 in *T. merianae* and from 0.75 to 0.99 in *T. rufescens* (Table 2).

**Table 2:** Partitioning of the variance in sperm morphometric and dynamic traits into: variance between species, among males of a single species, and within males

	Source of variance							
	Between species	Among males	Within male	Total	Repeatability *T. merianae*	Measurement error *T. merianae*	Repeatability *T. rufescens*	Measurement error *T. rufescens*
Head length								
Variance estimate ± SD	0.048 ± 0.221	0.735 ± 0.857	1.092 ± 1.045	1.875	0.97	0.06	0.97	0.05
Midpiece length								
Variance estimate ± SD	0.0269 ± 0.164	0.100 ± 0.316	0.297 ± 0.545	0.424	0.84	0.05	0.75	0.04
Flagellum length								
Variance estimate ± SD	2.582 ± 1.607	1.235 ± 1.111	12.163 ± 3.487	15.98	0.96	0.2	0.98	0.13
Total length								
Variance estimate ± SD	4.123 ± 2.030	1.755 ± 1.325	13.782 ± 3.712	19.66	0.97	0.23	0.99	0.14
VCL								
Variance estimate ± SD	4.309 ± 2.076	46.442 ± 6.815	53.01 ± 7.281	103.761	0.97	1.6	0.96	1.64
VSL								
Variance estimate ± SD	2.726 ± 1.651	34.473 ± 5.871	74.078 ± 8.607	111.269	0.99	0.61	0.99	0.68
LIN								
Variance estimate ± SD	0 ± 0	0.0014 ± 0.038	0.021 ± 0.1436	0.0224	0.97	0.00082	0.92	0.0011

The variance due to measurement errors was obtained using 15 males of each species; five spermatozoa of each male were measured four times. All morphometric and dynamic traits showed high and significant measurement repeatability (Sperm morphometric traits: *T. merianae* all *F*_74, 300_ > 24.47, *P* < 0.0001; *T. rufescens* all *F*_74, 300_ > 13.32, *P* < 0.0001; sperm dynamic traits: *T. merianae* all *F*_74, 300_ > 119, *P* < 0.0001; *T. rufescens* all *F*_74, 300_ > 46.14, *P* < 0.0001).

To explore differences in within-male variation in sperm traits among males, the likelihood ratio test was performed in each species separately, and the null hypothesis that intra-individual variance in sperm traits is the same among individuals was rejected (Table 3, Figs. 2, 3). Moreover, we studied the temporal variation of within-male variability in sperm traits. In *T. merianae,* we did not find differences among months during the reproductive period (Table 4). However, in *T. rufescenes,* we found differences in within-male variation of head length and total sperm length among months, with variation being higher in the middle of the season (November) (Table 4).

**Table 3:** Differences in within-male variation in sperm morphometric and dynamic traits among males in *Tupinambis merianae* and *T. rufescens*

	*T. merianae*		*T. rufescens*	
Sperm traits	L. ratio	*P*	L. ratio	*P*
Head length (*μ*m)	616.348	0.0001	322.689	0.0001
Midpiece length (*μ*m)	338.157	0.0001	226.093	0.0001
Flagellum length (*μ*m)	937.725	0.0001	728.798	0.0001
Total sperm length (*μ*m)	869.115	0.0001	816.444	0.0001
Curvilinear velocity (*μ*m/sec)	666.146	0.0001	454.042	0.0001
Straight velocity (*μ*m/sec)	721.528	0.0001	474.609	0.0001
Linearity	398.053	0.0001	131.623	0.0001

**Table 4:** Temporal variation in within-male variability in sperm traits of *Tupinambis*

		Reproductive period				
Species	Sperm traits	October	November	December	Statistic	*P*
*T. merianae* (*n* = 72)	CV Head length		7.30 ± 2.12	6.99 ± 2.28	0.76	0.3827
	CV Midpiece length		9.81 ± 1.69	9.61 ± 2.36	0.25	0.6162
	CV Flagellum length		5.59 ± 1.65	5.15 ± 2.17	1.11	0.2923
	CV Total sperm length		4.37 ± 1.22	4.11 ± 1.16	0.41	0.5209
*T. rufescens* (*n* = 41)	CV Head length	8.35 ± 3.29	8.87 ± 2.62	6.38 ± 1.76	12.68	0.0018
	CV Midpiece length	12.67 ± 2.47	11.58 ± 2.28	10.82 ± 2.08	3.77	0.1520
	CV Flagellum length	4.58 ± 1.96	6.29 ± 2.64	5.50 ± 2.84	3.31	0.1908
	CV Total sperm length	3.58 ± 1.44	5.49 ± 2.77	3.82 ± 1.42	6.57	0.0374

Finally, the comparison of CV of sperm morphometric traits showed that among males variation in head and midpiece length was higher in *T. rufescens*, whereas within males *T. rufescens* also had greater sperm variation of midpiece length than *T. merianae*. However, within-male variation in sperm dynamic traits was the same for both species (Table 1).

**Figure 4:**
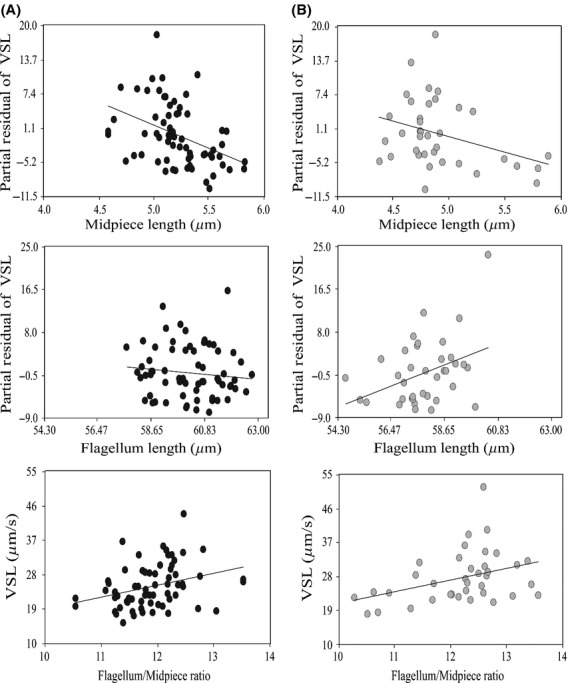
Relationship between sperm straight line velocity and sperm morphometric traits in *Tupinambis*. (A) *Tupinambis merianae*; (B) *Tupinambis rufescens*. Similar slopes between species were found (Dummy variable midpiece *F* = 0.48; *P* = 0.6204; flagellum: midpiece ratio *F* = 0.00062, *P* = 0.9803).

The relationship between sperm length and sperm velocity was evaluated in both species. We found that straight line velocity was negatively related to midpiece length and positively related to flagellum: midpiece ratio, with similar slopes between species (Table 5, Fig. 4). In addition, in *T. rufescens,* sperm velocity was positively related to flagellum length; this relationship was not found in *T. merianae* (Table 5). Moreover, we observed a positive relationship between within-male variation in head length and straight line velocity in *T. merianae* (slope: 0.81; *F*_1,62_: 4.91; *P* = 0.0304). By contrast, in *T. rufescens,* we found a negative relationship between within-male variation in midpiece length and sperm velocity (slope: −1.36; *F*_1,33_: 7.48; *P* = 0.01). Because linearity was high, more than 78% for both species (Mortimer 1997), similar results were found when we used curvilinear velocity (Tables S1, S2, Fig. S1).

**Table 5:** Relationship between sperm straight line velocity and sperm morphometric traits in *Tupinambis*

Species	Dependent variable	Predictor	Slope	*F*	*P*
*T. merianae* (*n =* 66)	VSL (*μ*m/sec)	Head length (*μ*m)	−1.17	0.01	0.9196
		Midpiece length (*μ*m)	−8.61	11.82	0.0011
		Flagellum length (*μ*m)	−0.45	0.63	0.4300
		Flagellum: head ratio	−0.06	0.000052	0.9819
		Flagellum:midpiece ratio	2.8	7.35	0.0086
		Head: midpiece ratio	5.59	3.54	0.0545
*T. rufescens* (*n* = 37)	VSL (*μ*m/sec)	Head length (*μ*m)	−0.59	4.00E-02	0.8388
		Midpiece length (*μ*m)	−5.63	4.93	0.0334
		Flagellum length (*μ*m)	1.99	5.1	0.0307
		Flagellum: head ratio	0.31	0.03	0.8752
		Flagellum: midpiece ratio	2.76	5.75	0.022
		Head: midpiece ratio	3.59	1.16	0.289

## Discussion

This study attempts to fill part of the striking lack of knowledge on the link between sperm morphometric and dynamic traits in the context of sperm competition risk in lizards. Sperm traits showed substantial variation at all levels examined: between species, among males within species, and within the ejaculate of individual males. Interestingly, in both *Tupinambis* species sperm velocity was found to be positively correlated with flagellum: midpiece ratio, with relatively longer flagella associated with faster sperm.

Because measuring postcopulatory sexual selection in wild animal populations is difficult, we inferred the risk of sperm competition based on reliable indicators of competition pressures. Here, we found differences in relative testis mass between *T. merianae* and *T. rufescens*. Moreover, Naretto et al. (2014) presented differences in the biased of sex ratio between these two species. The sexual proportion of individuals is often used as a predictor of the intensity of competition for mates, because it describes the relative number of males and females that are ready to mate (Kvarnemo and Simmons 2013). Then, these evidences suggest that, although they are sister species, they are under different competition pressures. Hence, if these species differed in competition context, we could expect differences in sperm traits. Here, we found that males of *T. merianae* present longer sperm than males of *T. rufescens*. Surprisingly, *T. rufescens* presented higher among-male sperm variation in head and midpiece length, higher within-male variation in midpiece length, and higher sperm velocity than *T. merianae* males.

Male reproductive success is determined by the interaction between the ability to access and choose females of the highest reproductive quality and the ability to outcompete the ejaculates of rival males (Cornwallis and Birkhead 2007; Keogh et al. 2013). Because sperm production is costly (Olsson et al. 1997), individual males may adjust the investment to maximize net reproductive benefit according to their mating role (Rudolfsen et al. 2006; Cornwallis and Birkhead 2007; Locatello et al. 2007), the number and quality of available females (Lüpold et al. 2011, 2012), and the risk and intensity of sperm competition (Parker 1998; Cornwallis and Birkhead 2007; Pizzari and Parker 2009; Kvarnemo and Simmons 2013). Accordingly, males of *T. merianae* and *T. rufescens* may produce spermatozoa of variable sperm size and velocity. Furthermore, we found an important within-male variation for all sperm traits measured. Males may produce a variety of sperm of different sizes as a strategy to maximize their fertilization success in a context of sperm competition (Helfenstein et al. 2010; Calhim et al. 2011). Moreover, because testis size changes throughout the breeding season, sperm morphology may vary within males during this period. This hypothesis would predict highly variable sperm at the beginning and the end of the season, when the testes are not in full breeding condition (Cramer et al. 2013). However, we found higher within-male variability in head length and total sperm length in the middle of the reproductive season (November), at the peak of maximum development of testis in *T. rufescens*, than at the beginning and the end of breeding season. These results suggest that a male may produce a mix of different sperm within a single ejaculate; this is important, because each component of spermatozoa may contribute with different functions and there might be trade-offs among functions (Pizzari and Parker 2009; Helfenstein et al. 2010; Bakker et al. 2014). Different sperm phenotypes may have advantages at different stages of the fertilization process (Bakker et al. 2014).

Different hypotheses have been proposed to explain the importance of different sperm components for sperm function; for example, a positive contribution of flagellum length to sperm velocity has been reported for several taxa (Gomendio and Roldan 2008; Fitzpatrick et al. 2009; Lüpold et al. 2009a; Tourmente et al. 2011a), increasing the thrust needed to propel sperm forward (Katz and Drobnis 1990). An increase of midpiece length may reflect sperm power output (Cardullo and Baltz 1991). An increase in the energetic reserves may increase longevity (Parker and Begon 1993). Finally, an elongation of head size may play an important role during sperm storage, contributing to sperm–female interactions (Pitnick et al. 2009b), and reducing the drag experienced by the sperm cell, which produces an increase in sperm swimming velocity (Malo et al. 2006). Recent studies suggest that intra-male variation in sperm traits could also mask length–speed relationships, because when average values for sperm length and speed are used, within-male variation is concealed (Simpson et al. 2013). By measuring multiple morphological traits for individual sperm cells and accounting for intra-male variation, length–speed relationships are more common than currently thought (Simpson et al. 2013). However, here, in both *Tupinambis* species, we found a negative relationship between sperm midpiece length and straight line velocity as well as higher swimming velocity in spermatozoa with longer flagellum relative to their midpiece. Moreover, in *T. rufescens,* we found a positive relationship between flagellum length and straight line velocity, which was not found in *T. merianae*. One possible explanation for this difference between species may be differences in the competition context to which they are exposed.

We know the importance of the relationship between sperm traits and fertilization efficiency to understand sperm evolution; however, elucidating this relationship in wild lizard populations of these species is difficult. However, several studies in different taxa have proposed a positive relationship between sperm velocity and male reproductive success under sperm competition (Birkhead et al. 1999; Gage et al. 2004; Gomendio and Roldan 2008). If sperm performance was related to within-male variability in sperm morphometry, we would expect a positive relationship between sperm velocity and within-male variability. When we tested this relationship, we found a positive weak relationship between VSL and within-male variability of head length in *T. merianae* and a negative relationship between VSL and within- male variability of midpiece length in *T. rufescens*. These results could be explained by the fact that sperm size may covary not only with velocity but also with other sperm performance parameters, such as longevity (Crean and Marshall 2008; Helfenstein et al. 2010). The theory predicts that sperm swimming speed will increase with increasing risk of sperm competition at the expense of the duration of motility (Ball and Parker 1996). Helfenstein et al. (2010) showed that sperm with long flagellum relative to their head swim faster than sperm with short flagellum, whereas the latter live longer than the former. As the midpiece is the main component providing sperm energy to move and survive in the female tract, considering our results it would be interesting to test whether midpiece contributes to sperm survival in these species. This is important in squamatas, which have been shown to store sperm for long periods (Birkhead and Møller 1993; Olsson and Madsen 1998; Holt and Loyd 2010). In particular, in many lizard species, females have special structures for storing sperm in their reproductive tracts (Sever and Hamlett 2002), and may store spermatozoa for at least some weeks between mating and ovulation (Olsson and Madsen 1998; Keogh et al. 2013). The time between insemination and egg encounter may influence a trade-off between velocity and longevity (Ball and Parker 1996).

In conclusion, our results show in lizards, the association between sperm length and function and suggest that the variability of sperm traits among and within males of a single species might be a strategy of sperm investment in lizards. The results also highlight the importance of studying within-male variability of sperm traits to understand the mechanism underlying sperm evolution.

## Supporting Information

**Table S1.** Relationship between sperm curvilinear velocity and sperm morphometric traits in *Tupinambis*. **Table S2.** Relationship between sperm curvilinear velocity and within-male variability in sperm morphometric traits.

**Figure S1**. Relationship between curvilinear sperm velocity and sperm morphometric traits in *Tupinambis*.
