# Supplementary material for: Variability in sperm form and function in the context of sperm competition risk in two Tupinambis lizards
Source: Ecol Evol. 2014 Oct 7;4(21):4080–92. doi: 10.1002/ece3.1262 (PMC4242561; doi:10.1002/ece3.1262)
Supplement: Supplementary file 1 [file ece30004-4080-SD1.docx]

**Table S1.** Relationship between sperm curvilinear velocity and sperm morphometric traits in *Tupinambis*

| Species | Dependent variable | Predictor | Slope | F | P |
| --- | --- | --- | --- | --- | --- |
| *T. merianae (n=66)* | VCL (µm/s) | Head length (µm) | -1.86 | 0.02 | 0.8803 |
|  |  | **Midpiece length (µm)** | **-10.99** | **14.58** | **0.0003** |
|  |  | Flagellum length (µm) | -0.62 | 0.94 | 0.3366 |
|  |  | Flagellum: head ratio | 0.74 | 0.06 | 0.806 |
|  |  | **Flagellum:midpiece ratio** | **3.58** | **8.89** | **0.0041** |
|  |  | Head:midpiece ratio | 6.41 | 3.36 | 0.0714 |
| *T. rufescens (n= 37)* | VCL (µm/s) | Head length (µm) | -0.92 | 3.00E-07 | 0.9996 |
|  |  | **Midpiece length (µm)** | **-6.86** | **5.52** | **0.0249** |
|  |  | Flagellum length (µm) | 2.01 | 4.04 | 0.0526 |
|  |  | Flagellum: head ratio | 0.28 | 0.02 | 0.8984 |
|  |  | **Flagellum:midpiece ratio** | **3.05** | **5.33** | **0.027** |
|  |  | Head:midpiece ratio | 3.64 | 0.91 | 0.3463 |

**Table S2.** Relationship between sperm curvilinear velocity and within-male variability in sperm morphometric traits.

| Species | Dependent variable | Predictor | Slope | F | p |
| --- | --- | --- | --- | --- | --- |
| *T. merianae (n=66)* | VCL (µm/s) | CV Head length (µm) | 0.77 | 3.02 | 0.0873 |
|  |  | CV Midpiece length (µm) | -0.41 | 0.93 | 0.3386 |
|  |  | CV Flagellum length (µm) | 0.08 | 0.03 | 0.8531 |
| *T. rufescens (n= 37)* | VCL (µm/s) | CV Head length (µm) | 0.24 | 0.07 | 0.7976 |
|  |  | **CV Midpiece length (µm)** | **-1.72** | **9.67** | **0.0039** |
|  |  | CV Flagellum length (µm) | -0.26 | 0.34 | 0.5628 |
